# Clinical profile and prognosis of elderly patients with left ventricular thrombus after anticoagulation

**DOI:** 10.1186/s12959-023-00520-4

**Published:** 2023-07-10

**Authors:** Qian Zhang, Zhongfan Zhang, Haikuo Zheng, Chengbing Wang, Miao Yu, Daoyuan Si, Wenqi Zhang

**Affiliations:** 1 Department of Cardiology, China-Japan Union Hospital of Jilin University, Jilin Provincial Molecular Biology Research Center for Precision Medicine of Major Cardiovascular Disease, Xiantai Street NO. 126, 130000 Changchun, Jilin China; 2 Department of Neurology, China-Japan Union Hospital of Jilin University, Changchun, Changchun, Jilin China

**Keywords:** Left ventricular thrombus, Elderly patients, Prognosis, Anticoagulation

## Abstract

**Background:**

Contemporary data regarding the clinical characteristics and prognosis of left ventricular thrombus (LVT) in older adults (aged ≥ 65 years old) are lacking. In this study, we characterized elderly patients with LVT (aged ≥ 65 years old) and investigated the long-term prognosis in this highly vulnerable patient population.

**Methods:**

This single-center, retrospective study was conducted from January 2017 to December 2022. Patients with a reported LVT were assessed primarily by transthoracic echocardiography (TEE) and classified into two groups: elderly LVT groups and younger LVT groups. All patients were treated with anticoagulant treatment. Major adverse cardiovascular event (MACE) was defined as the composite of all-cause mortality, systemic embolism, and rehospitalization for cardiovascular events. Survival analyses were performed with the Kaplan-Meier method and Cox proportional-hazard model.

**Results:**

A total of 315 eligible patients were included. Compared to the younger LVT group (n = 171), the elderly LVT group (n = 144) had a lower proportion of males and lower serum creatinine clearance, as well as a higher level of NT-proBNP, and a higher rate of history of systemic embolism. LVT resolution occurred in 59.7% and 69.0% of patients in the elderly LVT group and younger LVT group, respectively, with no significant difference (adjusted HR, 0.97; 95% CI, 0.74–1.28; P = 0.836). Yet, elderly patients with LVT, had higher prevalence rates of MACE (adjusted HR, 1.52; 95% CI, 1.10–2.11; P = 0.012), systemic embolism (adjusted HR, 2.81; 95% CI, 1.20–6.59; P = 0.017) and all-cause mortality (adjusted HR, 2.20; 95% CI, 1.29–3.74; P = 0.004) compared with younger patients with LVT. After adjusting for mortality in the Fine–Gray model, similar results were observed. Additionally, patients treated with different anticoagulation therapies (DOACs vs. warfarin) achieved a similar improvement in prognosis (P > 0.05) or LVT resolution (P > 0.05) in elderly patients with LVT.

**Conclusions:**

Our results found that elderly patients experiencing LVT have a poor prognosis compared with the younger ones. Clinical prognosis in elderly patients did not significantly differ with the type of anticoagulant used. With aging societies worldwide, further evidence of antithrombotic therapy in elderly individuals with LVT is necessary.

**Supplementary Information:**

The online version contains supplementary material available at 10.1186/s12959-023-00520-4.

## Background

The prevalence and complications of cardiovascular diseases have been increasing as the global population has aged. Among elderly individual, left ventricular thrombus (LVT) is associated with a higher risk of cardiovascular events and death [1, 2]. Due to the physiological changes associated with ageing, older people are generally exposed to a higher risk of thromboembolism. Furthermore, decreased hepatic metabolism and/or renal clearance predispose the individuals to drug-related complications [3]. Data from elderly patients suffering from atrial fibrillation (AF) indicate that the risk of bleeding during anticoagulation significantly increases with age [4]. Moreover, a subgroup analysis from the ROCKET AF study demonstrated that elderly patients have a higher stroke and major bleeding prevalence than younger patients during anticoagulation [5]. Yet, to date, no consensus or recommendation has been proposed for antithrombotic therapy in elderly LVT patients, and no randomized controlled trials (RCTs) or real-world studies have been published to guide our decision-making in treating these elderly patients in clinical practice. In addition, data on the prognosis of older adults with LVT are lacking. Hence, for this specific population, routine care is based on evidence from LVT studies in other clinical settings [6, 7]. Yet, whether the evidence from these various clinical settings can be generalized to elderly LVT patients requires additional data support. Thus, physicians continue to face challenges in LVT management in elderly patients and are in need of relevant real-world data on the prognosis to reflect the current status of this population.

Herein, we designed a retrospective study to characterize elderly LVT patients, and investigate their clinical prognosis and determine the impact of different anticoagulation therapies (DOACs vs. warfarin) on their prognosis.

## Methods

### Research design and population

This single-center, retrospective study was conducted at the China-Japan Union Hospital of Jilin University between January 2017 and December 2022. LVT was assessed primarily by transthoracic echocardiography (TTE), and other imaging modalities were used as a supplement. All consecutive echocardiogram reports conducted at the hospital were analyzed using computerized searches. Patients with a reported LVT (regardless of underlying disease) were screened, and only patients with LVT confirmed by 2 independent experts were included. Patients who were not receiving anticoagulant therapy or were lost to follow-up were excluded from the study. LVT was defined as an echo-dense mass distinct from the underlying myocardium and adjacent to a hypokinetic or akinetic myocardial LV segment or aneurysm, a clear thrombus-blood interface was required, and the LVT had to be visible in at least 2 views throughout the cardiac cycle. Detailed definitions of LVT echocardiographic evaluation were presented in the Supplemental Appendix;

The study protocol was approved by the Ethical review board of China-Japan Union Hospital of Jilin University (20,220,506,022). Informed consent was obtained from all individual participants by telephone. Written consent was waived due to the retrospective study design and minimal risk. The study was conducted in accordance with the principles of the Declaration of Helsinki.

### Data acquisition and patient follow-up

Detailed data on clinical characteristics were acquired via the patients’ electronic medical records. The collected outcome data were the presence or absence of an event during each patient’s follow-up period, the event’s date, and the situation regarding the administration of the anticoagulant during the event. Events included mortality, systemic embolism, rehospitalization for cardiovascular events, and bleeding. Events assessments by interviews with treating physicians or patients at each hospital visit. In addition, patients whose review events have not been documented in the medical record were contacted individually by telephone for a final ascertainment.

### Anticoagulation regimen

Patients with LVT were treated with anticoagulants (DOACs or warfarin) according to current guidelines and patient preference after evaluation by treating physicians [8, 9]. The DOACs dose selected based on creatinine clearance, age, and body weight. For patients treated with warfarin, we titrated the International Normalized Ratio (INR) range to 2.0–3.0, and monitored monthly after the target INR had been reached. In addition to checked for drug interactions about those drugs listed in the 2018 EHRA recommendations [10] and package inserts, physician also assessed individually for bleeding risk, as appropriate, before anticoagulation therapy was initiated.

### Study outcomes and definitions

LVT status was defined as described in the previous study [11]. LVT resolution was defined as the disappearance of a previously seen echo-dense mass in the left ventricle upon repeat echocardiography at the last available follow-up visit. LVT persistence was defined as an increased thrombus dimension, stable thrombus, or partial resolution of the thrombus, as demonstrated on echocardiography at the last available follow-up visit.

During the observation period, we collected the following clinical outcomes: Major adverse cardiovascular event (MACE) was defined as a composite of all-cause mortality, rehospitalization for cardiovascular reasons, or systemic embolism. Systemic embolism was defined as a composite of ischemic stroke, transient ischemic attack, myocardial infarction, or acute peripheral arterial embolism. Major bleeding, clinically relevant non-major bleeding, and minor bleeding were defined according to the International Society on Thrombosis and Haemostasis (ISTH) criteria [12, 13]. More detailed procedures and definitions of endpoint events are available in the Supplementary Appendix.

### Statistical analyses

Continuous data were expressed as the mean ± SD or median (Q1, Q3) based on data distribution and were analyzed using the Student’s t test or Mann–Whitney U test. Categorical data are expressed as frequency, counts, and percentages and were analyzed using the Pearson chi-squared test or Fisher’s exact test, as appropriate. To reduce the effect of study setting and confounding variables on the study results, the following procedure was conducted: (1) clinical variables associated with the prognosis of LVT were carefully selected based on previous studies; (2) patients who had not yet documented a review event in the medical record were contacted individually by telephone to finalize the accuracy; (3) data collection was standardized with precise definitions for each clinical covariate and measure; (4) all endpoint events were reviewed by a clinical academic group independent of this study based on prespecified event definition criteria; and (5) statistical analyses were predefined including the handling of confounding variables, sensitivity analysis and exploratory analysis to assess the stability of the study results. Regarding adverse events, only death was considered for analysis as a confounding variable. Due to the specificity of the death endpoint, other outcomes were not observed once death occurred, which would generate competing risks affecting the observation of other nonfatal endpoints. Since other common adverse events did not affect the observation of other clinical endpoints, they were not considered as confounding variables in this study. The multivariate Cox proportional hazard model was adopted to calculate the hazard ratio (HR) and the corresponding 95% confidence interval (CI) and p value for the prevalence of a clinical event between the two groups. The multivariate model included variables considered a priori outcome confounders of LVT. Clinical covariates included in the multivariate Cox proportional hazard regression model for each outcome were shown in Table S11 in Supplementary Appendix. Kaplan–Meier curves were plotted to illustrate the cumulative incidence between the two groups over time, and survival was compared using the log-rank test. Moreover, considering the high risk of death in elderly populations, the Fine-Gray model was used for sensitivity analysis to evaluate the stability of non-mortality findings. Finally, exploratory analyses were undertaken using the Cox proportional hazard test for interactions between different subgroups. Statistical analyses were undertaken with SPSS 24.0 (IBM, Armonk, NY, USA) and R 4.1.1 (R Foundation for Statistical Computing, Vienna, Austria), with significance defined as P < 0.05.

## Results

### Patient characteristics

**Fig. 1:**
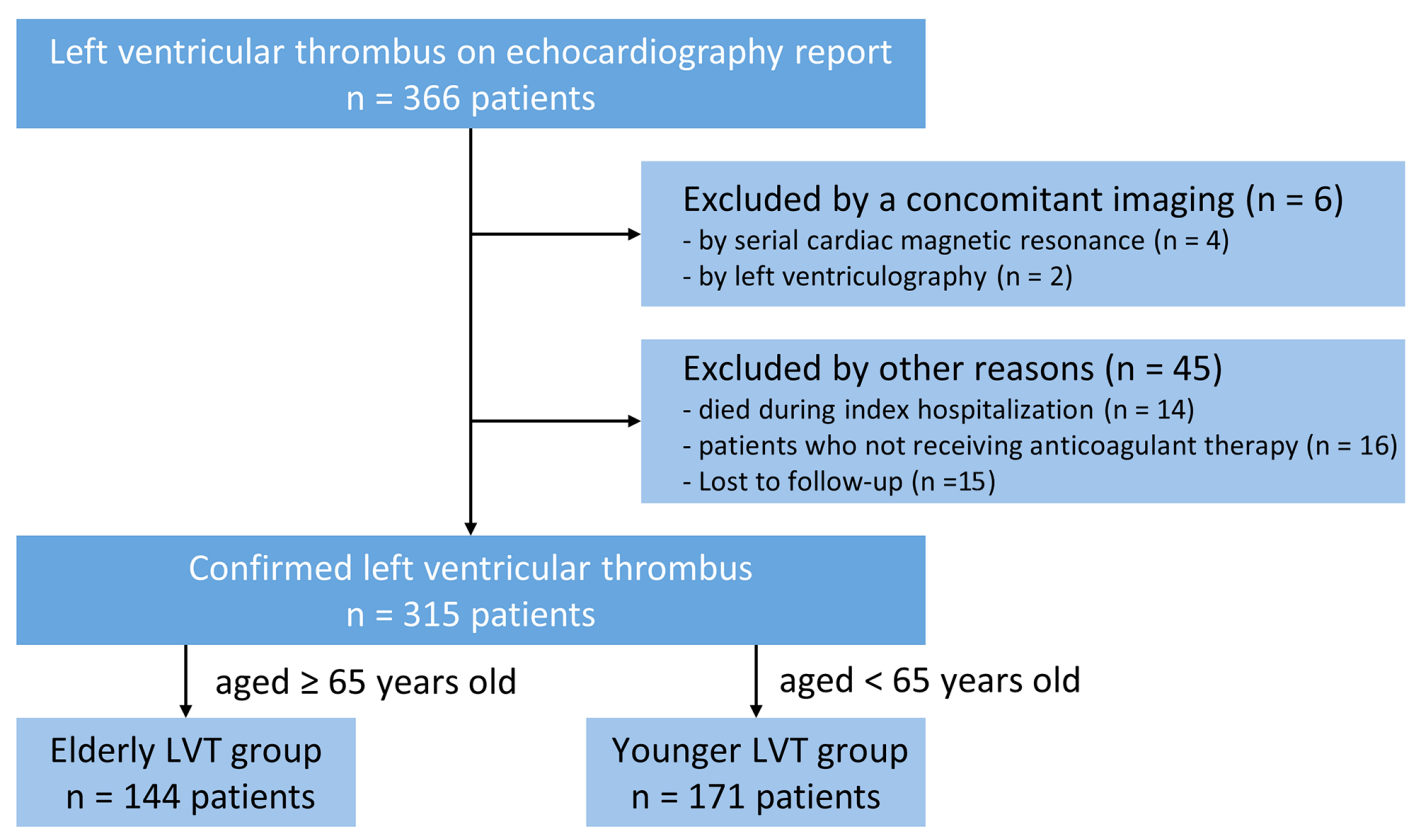
Flowchart of this study LVT: left ventricular thrombus

A total of 315 patients with LVT (mean age 60.8 ± 13.7 years) were enrolled in the study, and stratified according to age, 144 in the elderly LVT group and 171 in the younger LVT group (Fig. 1). The characteristics of patients according to age category at baseline are summarized in Table 1. Among these LVT patients, the majority (82.9%) presented with coronary artery disease (261 patients) (Table S1 in Supplementary Appendix). In the elderly group, these patients tended to have a lower proportion of males and lower serum creatinine clearance, a higher level of NT-proBNP, and a history of systemic embolism. No significant differences were found in the prevalence of hypertension, diabetes mellitus, LV aneurysm, LV ejection fraction, or thrombus size among the two age groups (Table 1). Of the 144 elderly patients with confirmed LV thrombus, 65 patients (45.1%) received warfarin, and 79 patients (54.9%) received DOACs therapy. A more detailed review of the study population, including the type of underlying disease and the type and dose of anticoagulant medications are shown in Table S1 - Table S4 in the Supplementary Appendix. Additional data on follow up, including duration of anticoagulation therapy and last TTE follow-up time, are included in Table S5 - Table S6 in the Supplement.

**Table 1:** Clinical characteristics according to age group

Characteristics baseline	Elderly LVT group (n = 144)	Younger LVT group (n = 171)	P value^a^
Age, years	71 (67, 76)	54 (47, 59)	0.001
Male, n (%)	102 (70.8)	142 (83.0)	0.010
Body mass index, kg/m^2^	23.7 (20.8, 26.2)	24.5 (22.5, 27.1)	0.034
Current smoker, n (%)	49 (34.0)	86 (50.3)	0.004
Hypertension, n (%)	61 (42.4)	66 (38.6)	0.497
Diabetes mellitus, n (%)	44 (30.6)	44 (25.7)	0.342
Atrial fibrillation, n (%)	13 (9.0)	9 (5.3)	0.192
Prior SSE, n (%)	50 (35.0)	31 (18.1)	0.001
Antiplatelet therapy	112 (77.8)	128 (74.9)	0.544
Creatinine clearance, mL/min/1.73 m^2^	68.3 (50.7, 85.5)	74.7 (58.5, 96.3)	0.011
Triglycerides, mmol/L	1.3(1.0-1.7)	1.3(1.0-1.8)	0.428
Cholesterol, mmol/L	4.2(3.7–4.9)	4.4(3.7-5.0)	0.212
NT-proBNP, pg/mL	5210 (2217.5, 10,775)	3900 (1210, 7624)	0.004
WBC, ×10^9^/L	7.7 (6.4, 9.9)	8.1 (6.5, 10.9)	0.257
Lymphocytes,×10^9^/L	1.6(1.2,2.4)	1.7(1.3,2.5)	0.735
MPV	10.0(9.2,10.8)	9.9(9.3,10.9)	0.917
Hemoglobin, g/L	139 (125.0, 152.8)	146 (132.0, 157.0)	0.983
D-dimer, mg/L	1.9 (0.7, 3.0)	1.9 (0.9, 3.0)	0.429
Fibrinogen, mg/dL	3.9 (3.1, 4.1)	3.4 (2.9, 4.0)	0.125
LV ejection fraction, %	38.3 (29.5, 46.9)	38.0 (28.0, 48.0)	0.871
LVEDD, mm	51.7 (47.2, 58.6)	55.6 (48.5, 61.5)	0.078
LV aneurysm, n (%)	33 (22.9)	34 (19.9)	0.512
Area of mitral regurgitation, cm^2^	3.5 (1.7, 5.6)	3.5 (2.0, 5.9)	0.977
Thrombus size, mm^2^	265 (140.3, 421.3)	313.0 (159.0, 485.0)	0.144
Medication			
Beta blockers, n (%)	116 (80.6%)	139 (81.3%)	0.869
ACEI, n (%)	96(66.7%)	105 (61.4%)	0.333
MRA, n (%)	75(52.1%)	81 (47.4%)	0.404
Duration of follow-up	23(10,35)	17(10,32)	-

^a^elderly LVT group vs. younger LVT group

SSE: stroke or systemic embolism; WBC: white blood cell; LV: left ventricular; LVEDD: left ventricular end-diastolic dimension; MPV: mean platelet volume; ACEI: angiotensin converting enzyme inhibitor; MRA: mineralcorticoid recept antagonist;

### LVT resolution

During follow-up (median: 19.0 months; IQR: 10.0–32.0 months), the overall rate of LVT resolution was 64.8%, with 87 patients (59.7%) in the elderly LVT group and 117 patients (69.0%) in the younger LVT group. There was no statistical difference in LVT resolution between the younger LVT group and elderly LVT group (adjusted HR: 0.97; 95%CI, 0.74–1.28; P = 0.836) (Table 2). After adjustment for mortality in the Fine–Gray model, similar results were observed, with no statistically significant difference in LVT resolution between the two groups (Gray’s test, P = 0.181) (Table S7 in Supplementary Appendix). Moreover, it has to be noted that LVT resolution was no statistically significant difference between patients treated with DOACs and those treated with warfarin (62.0% vs. 58.4%; HR: 1.19; 95%CI: 0.78–1.83; P = 0.425) in the elderly LVT group in our study (Table S10 in Supplementary Appendix).

**Table 2:** Outcomes of Cox proportional hazards regression analysis in LVT patients

	Events, no. (%)			
	Elderly LVT group	Younger LVT group	Adjusted HR (95% CI)	P value^a^
Outcomes	(n = 144)	(n = 171)		
LVT resolution	87 (59.7)	117 (69.0)	0.97 (0.74–1.28)	0.836
Major adverse cardiovascular events	88 (61.1)	68 (40.0)	1.52 (1.10–2.11)	0.012
All-cause mortality	50 (34.7)	23 (13.5)	2.20 (1.29–3.74)	0.004
Systemic embolism	19 (13.2)	8 (4.7)	2.81 (1.20–6.59)	0.017
Rehospitalization for cardiovascular events	43 (29.9)	41 (24.0)	1.37 (0.89–2.13)	0.156
Bleeding events	14 (9.7)	9 (5.3)	2.13 (0.68–5.50)	0.086
Major bleeding	4 (2.8)	3 (1.8)	—	—
CRNM bleeding	3 (2.1)	1 (0.6)	—	—
Minor bleeding	7 (4.8)	5 (3.0)	—	—

^a^elderly LVT group vs. younger LVT group

LVT: left ventricular thrombus; CRNM: clinically relevant non-major

### Major adverse cardiovascular events

**Fig. 2:**
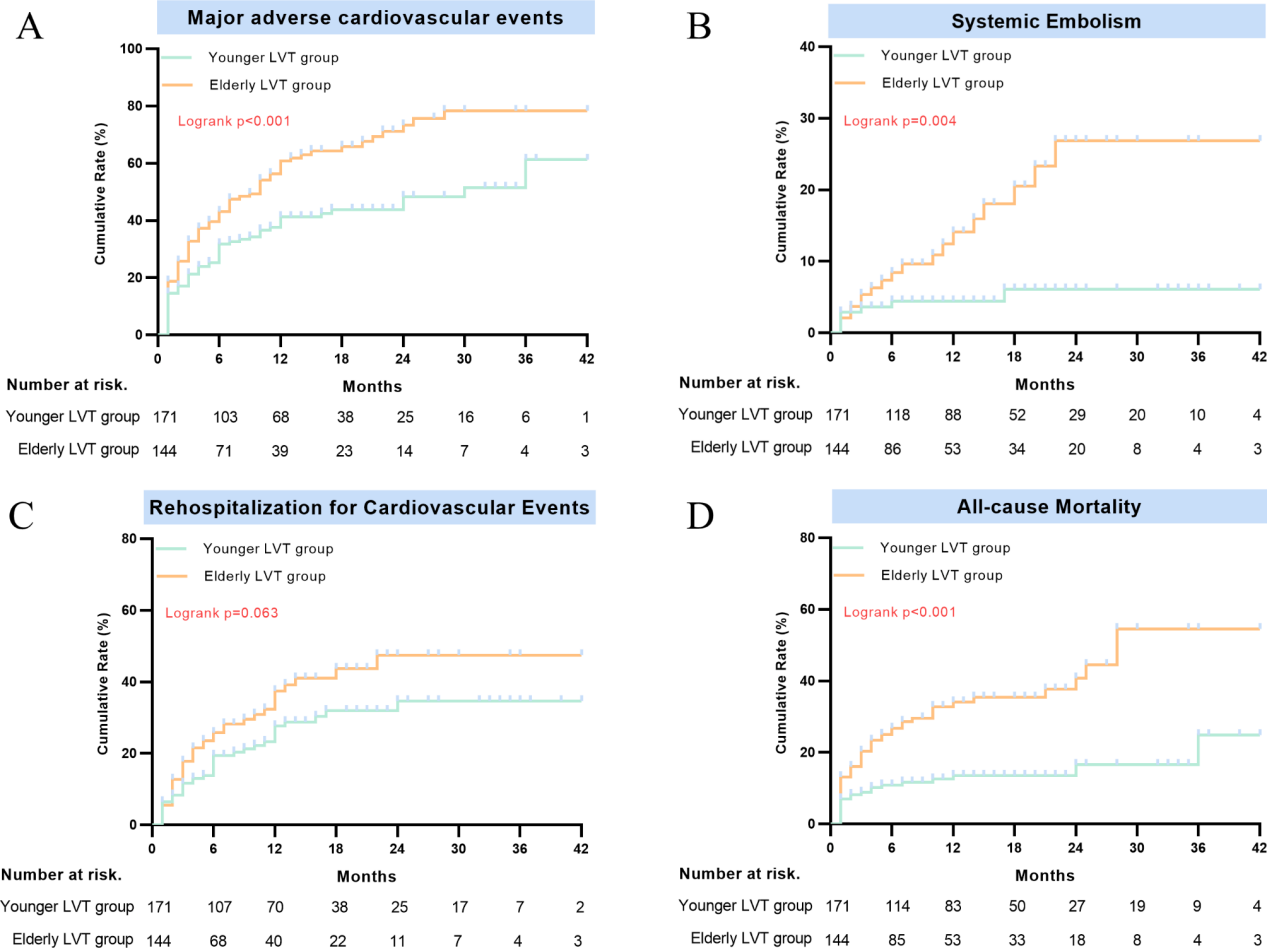
Kaplan–Meier curves for outcomes between two groups during a mean follow-up of 13 months. **(A)** Major adverse cardiovascular events, **(B)** Embolic complications, **(C)** Rehospitalization for cardiovascular events. **(D)** All-cause mortality

MACE occurred in 61.1% (n = 88) of patients in the elderly group and 40% (n = 68) of patients in the younger group. All-cause mortality occurred in 34.7% (n = 23) of patients in the elderly group and 13.5% (n = 50) in the younger group. On multivariable Cox proportional hazards regression analysis, the incidence of MACE (adjusted HR: 1.52; 95% CI, 1.10–2.11; P = 0.012; log-rank test, P < 0.001) and all-cause mortality (adjusted HR: 2.20; 95% CI, 1.29–3.74; P = 0.004; log-rank test, P < 0.001) were higher in elderly LVT group than the younger LVT group (Table 2; Fig. 2A, D). Furthermore, MACE (62.1% vs. 60.0%; HR: 1.10; 95% CI: 0.72–1.67; P = 0.664) and all-cause mortality (34.2% vs. 35.4%; HR: 0.99; 95% CI, 0.57–1.75; P = 0.998) were no statistically significant difference between patients treated with DOACs and those treated with warfarin in the elderly LVT group (Table S10 in Supplementary Appendix). There were 29.9% rehospitalization for cardiovascular events in the elderly group and 24.0% in the younger group (adjusted HR: 1.37; 95% CI: 0.89–2.13; P = 0.156; log-rank test, P = 0.068) (Table 2; Fig. 2C, Table S8 in Supplementary Appendix).

In terms of systemic embolism and bleeding events, the elderly group had a relatively higher prevalence of systemic embolism compared with the younger group (13.2% vs. 4.7%). The results were similar in the multivariate cox proportional hazard model, Fine–Gray model, and log-rank test (adjusted HR: 2.81; 95% CI: 1.20–6.59; P = 0.017; Gray’s test, P = 0.017; log-rank test, P = 0.004) (Table 2; Fig. 2B, Table S9 in Supplementary Appendix). In addition, a similar risk of bleeding events defined by ISTH criteria was observed between the elderly LVT group and younger LVT group (9.7% vs. 5.3%; adjusted HR: 2.13; 95% CI: 0.68–5.50; P = 0.086) (Table 2). Meanwhile, a similar prevalence of systemic embolism (12.6% vs. 13.8%; HR: 0.72; 95% CI: 0.86 (0.35–2.12); P = 0.744) and bleeding events were observed among patients treated with DOACs and warfarin (10.1% vs. 9.2%; HR, 0.97; 95% CI, 0.33–2.88; P = 0.953) (Table S10 in Supplementary Appendix).

### Exploratory analyses

In addition, we stratified subgroups according to sex, antiplatelet therapy, type of anticoagulation therapy, and LV ejection fraction for exploratory analysis. The results were consistent for most subgroups, with no significant interactions (Figure S1–S5 in Supplementary Appendix).

## Discussion

This study provides a relatively well-characterized contemporary cohort involving 144 elderly patients with LVT. We did not observe a statistically significant difference between elderly and younger LVT patients in terms of thrombus resolution. Yet, elderly patients with LVT had a higher risk of MACE, systemic embolism, and all-cause mortality compared with younger LVT patients. In addition, clinical outcomes in elderly patients did not significantly differ with the type of anticoagulant used (DOACs vs. warfarin). To the best of our knowledge, this is the first real-world study that investigated the prognosis of elderly patients with LVT.

In the present study, we observed that more than one-third of elderly patients did not achieve LVT resolution, which was similar to what has been reported in all LVT populations. A study from Europe examined 156 patients with all diseases complicated with LVT, the median follow-up time was 632 days, and the thrombus resolution rate was 66.7% [11]. Another study from China reported a thrombus resolution rate of 64.1% among 237 patients with all diseases complicated with LVT who were followed up for a median of 736 days [14]. In addition, we further observed no significant difference in the incidence of thrombus resolution in elderly patients treated with DOACs versus those treated with warfarin. These observations are consistent with some findings in other clinical settings [7, 15, 16]. However, some studies have shown that LVT resolution was independently associated with favorable long-term outcomes, such as reduced MACE and mortality [11, 17]. These results emphasize that treatment strategies for LVT should be further advanced to improve LVT resolution. Given that there are no specific guidelines for anticoagulation therapy in elderly LVT patients, randomized controlled trials (RCTs) are needed to focus on the specific effect of different anticoagulants on LVT resolution in the elderly populations.

With respect to clinical outcomes, we observed higher risk of MACE, systemic embolism, and all-cause mortality in elderly patients with LVT than in younger patients with LVT. Considering the high risk of death in older adults, the results remained consistent after excluding the risk of mortality by competitive risk analysis. Lattuca and colleagues showed that mortality and embolic complications occurred in 18.9% (n = 30) and 22.2% (n = 35) of all disease patients complicated with LVT [11]. Another study from East China showed that the mortality and the embolic complication rates were 28.3% and 13%, respectively [14]. In our study, 34.7% of patients died, and 13.2% had embolic complications in the elderly group, which was higher compared to the similar study mentioned above. This highlights that even in the contemporary era of primary PCI and the generalized use of dual antiplatelet therapy (DAPT), the prognosis of LVT in the elderly can still be devastating. The main reason for the increased risk of death in our study may be the increased risk of embolic complications, especially stroke and acute myocardial infarction turn out to be a direct cause of death in those patients. Improved regimens to accelerate LVT resolution should be considered to ameliorate such a poor prognosis, a factor associated with a reduced prevalence of death in a study [11]. Ageing also entails various challenges for antithrombotic therapy. The risk of bleeding increases with age and may be exacerbated by anticoagulants, as demonstrated in several RCTs [5, 18]. In our study, elderly patients seemed to have a higher tendency to bleed (9.7% vs. 5.3%), but there was no significant difference. Given the cohort size and the number of bleeding events, it is possible that our analysis was underpowered to enable the formation of solid conclusions about bleeding events. Therefore, our results on bleeding events should be interpreted with caution.

Ascertaining optimal medical therapy to reduce the complications of LVT in this highly vulnerable patient population is challenging. Several landmark RCTs evaluating DOACs compared with warfarin for LVT management in elderly patients with AF reported efficacy for preventing systemic embolism and accompanied by a lower risk of intracranial hemorrhage (major bleeding) [18–20]. In the ENGAGE AF-TIMI 48 trial and ARISTOTLE trial [19, 20], DOACs provided superior net clinical outcomes, and the prevalence of major bleeding was consistently lower with DOACs than with warfarin in elderly patients. Given these clinical benefits of DOACs, some guidelines consider DOACs a promising alternative to vitamin-K antagonists in LVT [21, 22], and they are being used increasingly as an off-label alternative. However, the treatment evidence using DOACs for LVT management is limited and controversial [23–26]. Only two RCTs have investigated DOACs vs. warfarin in patients with LVT. Both studies demonstrated that DOACs were not inferior to warfarin and that thrombosis resolved more rapidly [27, 28]. Given the small cohort size of those two studies and unblinded design, their results should be considered inconclusive. Furthermore, the largest available cohort study of LVT (n = 514) showed a higher risk of stroke and systemic embolism with DOACs [29]. However, due to its retrospective design, the absence of prognostic data beyond embolic events (bleeding, mortality, and LVT resolution), and the different types of DOACs, these results should also be interpreted with caution. Overall, the use of DOACs in LVT patients should continue to be investigated, and more data are needed to support decision-making. DOAC dosing recommendations for the treatment of LVT can be complex, and there are no clear guideline recommendations. Previous studies have shown that underdosing of DOACs can increase the risk of stroke, while inappropriate overdosing can increase the risk of bleeding [30–32]. Additionally, drug-drug interactions are numerous and may lead to dose variation. While this is not something we can determine from our data, it should be explored in future studies. We found that in elderly patients with LVT, clinical outcomes in elderly patients did not significantly differ with the type of anticoagulant used. These real-world data provide further insights into LVT management in elderly patients. Nevertheless, our findings should also be interpreted with caution due to the small sample size and retrospective design.

There are several limitations in this study. First, this was a retrospective observational study from a large tertiary referral center. This retrospective observational design means that despite efforts to adjust for confounding variables, there may still be residual confounders that we have been unable to correct in this study. Selection bias, including antithrombotic treatment strategies, may also limit the ability to make comparisons based on treatment outcomes. Second, LVT was identified by routine echocardiography. Although the study used the standardized expert assessment for echocardiograms, the sensitivity and specificity for detection may be lower compared to other imaging modalities such as CMR. Third, this study has a relatively small sample size. Although this is the largest contemporary series evaluating elderly patients with LVT, our results should be considered exploratory rather than conclusive. Finally, the prevalence of bleeding events in our study was so low that we could not identify a significant difference between the two groups. The low prevalence of major bleeding events in retrospective studies does not reflect their true prevalence in clinical practice, which may be related to patient selection, the intensity of monitoring, follow-up, or other factors.

## Conclusions

Our results found that elderly patients experiencing LVT have a poor prognosis compared with the younger ones. Clinical outcomes in elderly patients did not significantly differ with the type of anticoagulant used. With aging societies worldwide, further evidence of antithrombotic therapy in elderly individuals with LVT is necessary.

## Electronic supplementary material

Below is the link to the electronic supplementary material.

Supplementary Material 1

## Data Availability

The raw data supporting the conclusions of this article will be made available by the authors, without undue reservation.

## Abbreviations

LVT: left ventricular thrombus
MACE: Major adverse cardiovascular event
TTE: transthoracic echocardiography
INR: International Normalized Ratio
