# Supplementary material for: Clinical profile and prognosis of elderly patients with left ventricular thrombus after anticoagulation
Source: Thromb J. 2023 Jul 10;21:75. doi: 10.1186/s12959-023-00520-4 (PMC10332057; doi:10.1186/s12959-023-00520-4)
Supplement: Supplementary file 1 — Supplementary Material 1 [file 12959_2023_520_MOESM1_ESM.docx]

**Supplementary Appendix**

**Clinical outcomes of elderly patients with left ventricular thrombus**

Qian Zhang^1^, Zhongfan Zhang^1^, Haikuo Zheng^1^, Chengbing Wang^2^, Miao Yu^1^, Daoyuan Si^1*^, Wenqi Zhang^1*^

^1^ Department of Cardiology, China-Japan Union Hospital of Jilin University, Jilin Provincial Molecular Biology Research Center for Precision Medicine of Major Cardiovascular Disease, Changchun, Jilin, China;

^2^ Department of Neurology, China-Japan Union Hospital of Jilin University, Changchun, Jilin, China.

*These authors are both the corresponding authors.

Corresponding Author:

Wenqi Zhang

Xiantai Street NO.126, Changchun, Jilin, 130000, China

Email address: wenqi@jlu.edu.cn

Summary

[**1. Left Ventricular Thrombus Echocardiographic Evaluation** 3](#_Toc137460261)

[**1.1 Echocardiographic procedures to confirm LV thrombus** 3](#_Toc137460262)

[**1.2 Non-contrast echocardiography** 3](#_Toc137460263)

[**1.3 Contrast Echocardiography** 3](#_Toc137460264)

[**2. Endpoint Definition and Adjudication** 3](#_Toc137460265)

[**2.1 Endpoint adjudication procedure** 4](#_Toc137460266)

[**2.2 Definition of LVT resolution and persistence** 4](#_Toc137460267)

[**2.4 Definition of bleeding events** 5](#_Toc137460268)

[**3. Statistical plan: Adjustment for competing risk** 6](#_Toc137460269)

**Table S1:**[Underlying Disease of LVT 8](#_Toc137460270)

**Table S2:**[Most common underlying etiologies for LVT 8](#_Toc137460271)

**Table S3:**[High risk diseases for thrombus in All LVT Patients 8](#_Toc137460272)

**Table S4:**[Antithrombotic Strategy Following LVT Diagnosis 9](#_Toc137460273)

**Table S5:**[Median duration of anticoagulation therapy (median [IQR]) (Months) 9](#_Toc137460274)

**Table S6:**[Median time of last TTE follow-up (median [IQR]) (Months) 9](#_Toc137460275)

**Table S7:**[Competing Risks Analysis for LVT Resolution 10](#_Toc137460276)

**Table S8:**[Competing Risks Analysis for Rehospitalization for Cardiovascular Events 10](#_Toc137460277)

**Table S9:**[Competing Risks Analysis for Systemic Embolism 10](#_Toc137460278)

**Table S10:**[Outcomes of Cox proportional hazards regression analysis of Rivaroxaban *vs*. warfarin in the elderly group 11](#_Toc137460279)

**Table S11:**[clinical covariates included in the multivariate Cox proportional hazard regression model for each outcome 11](#_Toc137460280)

[**Figure S1:**Subgroup Analysis of LVT resolution 12](#_Toc137460281)

[**Figure S2:**Subgroup Analysis of Major Adverse Cardiovascular Events 12](#_Toc137460282)

[**Figure S3:**Subgroup Analysis of Rehospitalization for Cardiovascular Events 13](#_Toc137460283)

[**Figure S4:**Subgroup Analysis of Systemic Embolism 13](#_Toc137460284)

[**Figure S5:**Subgroup Analysis of All-cause Mortality 14](#_Toc137460285)

**1. Left Ventricular Thrombus Echocardiographic Evaluation**

**1.1 Echocardiographic procedures to confirm LV thrombus**

Echocardiograms were interpreted by two experienced echocardiographers. Echocardiographic data obtained from medical records or from patients or their family members. If there is disagreement between the echocardiographers 's views during the event review, the images are submitted to another echocardiographer for review to determine the final outcome.

**1.2 Non-contrast echocardiography**

The LVT observed by Non-contrast echocardiography was defined as follows:

- a dense echogenic mass in the left ventricular cavity with a structural texture different from that of the myocardium
- Clear demarcation toward the cavity
- Can be separated by the endocardium
- Visible throughout the cardiac cycle

**1.3 Contrast Echocardiography**

The LVT observed by contrast echocardiographic imaging was defined as follows:

- mass with a non-cloudy filling defect, as opposed to a cloudy LV cavity under contrast visible separation from the underlying endocardium;
- is visible throughout the cardiac cycle;
- Compared to tumors, LVT are avascular and do not show contrast enhancement after high MI contrast.

**2. Endpoint Definition and Adjudication**

**2.1 Endpoint adjudication procedure**

Follow‐up was acquired through personal interviews in routine visits, telephone contact with patients/families, and medical records. All event adjudications were performed by a clinical academic panel from the Jilin Cardiovascular Disease Research Center based on pre-specified event definition criteria and carefully assessed whether events occurring in the investigator were consistent with the intended endpoints in the protocol. The clinical academic panel consists of three experts with extensive clinical practice experience in internal medicine and cardiology who are unknown to the study. All patient identifying information was kept anonymous to ensure a fair event adjudication. During the review process, the study coordinator randomly assigned each endpoint to two independent experts for review; if consensus was reached after the review, the adjudication was considered complete, and in case of disagreement, another independent expert provided the final decision.

**2.2 Definition of LVT resolution and persistence**

LV thrombus status was defined as described in the previous study [1].

**LVT resolution** was defined as the disappearance of previously seen echo dense mass in the left ventricle upon repeat echocardiography at the last available follow-up visit.

**LVT persistence** was defined as increased thrombus dimension, stable thrombus, or partial resolution of the thrombus as demonstrated on echocardiography at the last available follow-up visit.

**2.3 Definition of major adverse cardiovascular events**

**Major adverse cardiovascular events (MACE)** were defined as a composite of all-cause mortality, rehospitalization for cardiovascular reasons, or systemic embolism.

**Systemic embolism** was defined as a composite of ischemic stroke, transient ischemic attack, myocardial infarction, or acute peripheral arterial embolism.

**Rehospitalization for cardiovascular events** was defined as hospitalization for worsening cardiovascular events.

**Ischemic Stroke** was defined as a rapid onset of a documented focal neurologic deficit lasting 24 h or until death, or if <24 h, there was a clinically relevant lesion on brain imaging. Patients with focal neurologic deficits secondary to brain trauma, tumour, infection, or other non-vascular cause were excluded [2].

**TIA** was defined as a documented episode(s) of focal neurologic deficit lasting between 30 s and 24 h, and without brain imaging consistent with a stroke [3].

**Acute limb ischemia** was defined as a documented extracranial arterial embolic episode manifesting as a sudden loss of perfusion of an organ or a limb. It included upper and lower extremity, renal, mesenteric, and abdominal aortic systems [4].

**2.4 Definition of bleeding events**

Bleeding events were classified as minor bleeding, clinically relevant non-major (CRNM) or major bleeding using the International Society on Thrombosis and Haemostasis (ISTH) definition [5, 6].

**Major bleeding** was defined as having a symptomatic presentation and:

1) Fatal bleeding, and/or

2)Bleeding in a critical area or organ, such as intracranial, intraspinal, intraocular, retroperitoneal, intra-articular or pericardial, or intramuscular with compartment syndrome, and/or

3)Bleeding causing a fall in hemoglobin level of 20 g /L (1.24 mmol /L) or more, or leading to transfusion of two or more units of whole blood or red cells

C**linically relevant nonmajor bleeding** was defined as：

Any sign or symptom of hemorrhage (e.g., more bleeding than would be expected for a clinical circumstance, including bleeding found by imaging alone) that does not fit the criteria for the ISTH definition of major bleeding but does meet at least one of the following criteria:

1. requiring medical intervention by a healthcare professional;
2. leading to hospitalization or increased level of care;
3. prompting a face to face (i.e., not just a telephone or electronic communication) evaluation.

**Minor bleeding** was defined as not meeting criteria for major or clinically relevant nonmajor.

**3. Statistical plan: Adjustment for competing risk**

When the study was initially designed, there was no pre-specified plan to adjust for the competing risk of mortality. But at the study end, consistent with the change in convention in the statistical methodology, it was decided that all primary analyses should be conducted after adjusting for the competing risk for mortality. Accordingly, for the endpoints (LVT resolution, Systemic embolism, Rehospitalization for cardiovascular events) the data was calculated after adjusting for mortality. In this Supplementary material, as indicated for Supplementary Table S3-S5.

**References**

1. Lattuca B, Bouziri N, Kerneis M, Portal JJ, Zhou J, Hauguel-Moreau M, Mameri A, Zeitouni M, Guedeney P, Hammoudi N *et al*: Antithrombotic Therapy for Patients With Left Ventricular Mural Thrombus. *J Am Coll Cardiol* 2020, 75(14):1676-1685.
2. Polak JF, Sacco RL, Post WS, Vaidya D, Arnan MK, O'Leary DH: Incident stroke is associated with common carotid artery diameter and not common carotid artery intima-media thickness. *Stroke* 2014, 45(5):1442-1446.
3. Hicks KA, Tcheng JE, Bozkurt B, Chaitman BR, Cutlip DE, Farb A, Fonarow GC, Jacobs JP, Jaff MR, Lichtman JH *et al*: 2014 ACC/AHA Key Data Elements and Definitions for Cardiovascular Endpoint Events in Clinical Trials: A Report of the American College of Cardiology/American Heart Association Task Force on Clinical Data Standards (Writing Committee to Develop Cardiovascular Endpoints Data Standards). *Circulation* 2015, 132(4):302-361.
4. Bekwelem W, Connolly SJ, Halperin JL, Adabag S, Duval S, Chrolavicius S, Pogue J, Ezekowitz MD, Eikelboom JW, Wallentin LG *et al*: Extracranial Systemic Embolic Events in Patients With Nonvalvular Atrial Fibrillation: Incidence, Risk Factors, and Outcomes. *Circulation* 2015, 132(9):796-803.
5. Schulman S, Kearon C, Subcommittee on Control of Anticoagulation of the S, Standardization Committee of the International Society on T, Haemostasis: Definition of major bleeding in clinical investigations of antihemostatic medicinal products in non-surgical patients. *J Thromb Haemost* 2005, 3(4):692-694.
6. Kaatz S, Ahmad D, Spyropoulos AC, Schulman S, Subcommittee on Control of A: Definition of clinically relevant non-major bleeding in studies of anticoagulants in atrial fibrillation and venous thromboembolic disease in non-surgical patients: communication from the SSC of the ISTH. *J Thromb Haemost* 2015, 13(11):2119-2126.

**Table S1:**

Underlying Disease of LVT

| **Underlying Disease causing Heart Failure** | | |
| --- | --- | --- |
| **Underlying disease** | **Number of patients, n** | **Number of patients, %** |
| **Coronary artery disease** | 261 | 82.9% |
| **Dilated cardiomyopathy** | 25 | 7.92% |
| **Hypertrophic cardiomyopathy** | 1 | 0.32% |
| **Myocarditis** | 7 | 2.22% |
| **Valvular heart disease** | 10 | 3.17% |
| **Congenital heart disease** | 2 | 0.62% |
| **Others** | 9 | 2.85% |
| **Total** | 315 | 100% |

**Table S2:**

Most common underlying etiologies for LVT

| **Most common underlying etiologies for LVT** | | |
| --- | --- | --- |
|  | **Elderly LVT group** | **Younger LVT group** |
| **MI (within 3 month)** | 105(72.9%) | 133 (77.7%) |
| **LV failure** | 127 (88.2%) | 145 (84.8%) |

**Table S3:**

High risk diseases for thrombus in All LVT Patients

| **High risk diseases for thrombus in All LVT Patients** | | |
| --- | --- | --- |
|  | **Elderly LVT group** | **Younger LVT group** |
| **active malignancy** | 3（2.1%） | 2（1.2%） |
| **connective tissue disease** | 2（1.4%） | 3（1.8%） |
| **Antiphospholipid antibodies** | 0 | 0 |
| **inherited thrombophilia** | 0 | 0 |

**Table S4:**

Antithrombotic Strategy Following LVT Diagnosis

| **Antithrombotic Strategy in All LVT Patients** | | | |
| --- | --- | --- | --- |
|  | **Elderly LVT group** | **Younger LVT group** |  |
| **No Antiplatelet** | 32 (22.2%) | 43 (25.1%) |  |
| **Anticoagulation + antiplatelet therapy** | 38 (26.4%) | 32 (18.7%) |  |
| **Anticoagulation + dual antiplatelet therapy** | 74 (51.4%) | 98 (57.3%) |  |
| **Anticoagulation type** |  |  |  |
| **Vitamin K antagonist** | 65 (45.1%) | 45 (26.3%) |  |
| **Direct oral anticoagulant** | 79 (54.9%) | 126 (73.7%) |  |
| **Rivaroxaban 10mg** | 4（5.1%） | 7（5.6%） |  |
| **Rivaroxaban 15mg** | 45（57.0%） | 73（57.9%） |  |
| **Rivaroxaban 20mg** | 28（35.4%） | 43（34.1%） |  |
| **Rivaroxaban 10mg** | 4（5.1%） | 7（5.6%） |  |

**Table S5:**

Median duration of anticoagulation therapy (median [IQR]) (Months)

| **Median duration of anticoagulation therapy (median [IQR]) (Months)** | | |
| --- | --- | --- |
|  | **Elderly LVT group** | **Younger LVT group** |
| **Warfarin** | 5 (1,11) | 4 (1,10) |
| **DOACs** | 4 (1,10) | 4 (1,9) |

IQR indicates interquartile range.

**Table S6:**

Median time of last TTE follow-up (median [IQR]) (Months)

| **Median time of last TTE follow-up (median [IQR]) (Months)** | | |
| --- | --- | --- |
|  | **Elderly LVT group** | **Younger LVT group** |
| **warfarin** | 10（4,18） | 10（3,16） |
| **DOACs** | 14（5,21） | 10(4,19) |

IQR indicates interquartile range.

**Table S7:**

Competing Risks Analysis for LVT Resolution

| **Rehospitalization for LVT Resolution- Cumulative incidence function (CIF)** | | | | | | | |  |
| --- | --- | --- | --- | --- | --- | --- | --- | --- |
|  | **6M** | **12M** | **18M** | **24M** | **30M** | **36M** | **42M** | **Gray’s test p value** |
| Younger LVT group | 43.5% | 71.9% | 81.4% | 84.7% | 87.0% | 87.0% | 87.0% | 0.181 |
| Elderly LVT group | 38.3% | 66.3% | 74.1% | 78.5% | 82.8% | 82.8% | 82.8% |  |

**Table S8:**

Competing Risks Analysis for Rehospitalization for Cardiovascular Events

| **Rehospitalization for Cardiovascular Events- Cumulative incidence function (CIF)** | | | | | | | |  |
| --- | --- | --- | --- | --- | --- | --- | --- | --- |
|  | **6M** | **12M** | **18M** | **24M** | **30M** | **36M** | **42M** | **Gray’s test p value** |
| Younger LVT group | 17.6% | 25.9% | 30.2% | 32.9% | 32.9% | 32.9% | 32.9% | 0.068 |
| Elderly LVT group | 24.2% | 34.2% | 40.4% | 44.2% | 44.2% | 44.2% | 44.2% |  |

**Table S9:**

Competing Risks Analysis for Systemic Embolism

|  | **Systemic Embolism- Cumulative incidence function (CIF)** | | | | | | | | |
| --- | --- | --- | --- | --- | --- | --- | --- | --- | --- |
|  | | **6M** | **12M** | **18M** | **24M** | **30M** | **36M** | **42M** | **Gray’s test p value** |
| Younger LVT group | | 4.4% | 4.4% | 4.4% | 4.6% | 18.1% | 18.1% | 18.1% | 0.017 |
| Elderly LVT group | | 5.8% | 11.6% | 18.2% | 24.5% | 24.5% | 24.5% | 24.5% |  |

**Table S10:**

Outcomes of Cox proportional hazards regression analysis of Rivaroxaban *vs*. warfarin in the elderly group

| **Clinical Endpoint** | **DOACs**  **(N = 79)** | **Warfarin**  **(N = 65)** | **Rivaroxaban**  **vs. warfarin**  **HR (95% CI)** | **P value^a^** |
| --- | --- | --- | --- | --- |
| LVT resolution | 49 (62.0) | 38 (58.4) | 1.19 (0.78–1.83) | 0.425 |
| Major adverse cardiovascular events | 49 (62.1) | 39 (60.0) | 1.10 (0.72–1.67) | 0.664 |
| All-cause mortality | 27 (34.2) | 23 (35.4) | 0.99 (0.57–1.75) | 0.998 |
| Systemic embolism | 10 (12.6) | 9 (13.8) | 0.86 (0.35–2.12) | 0.744 |
| Rehospitalization for cardiovascular events | 27 (34.2) | 16 (24.6) | 1.48 (0.80–2.75) | 0.213 |
| Bleeding events | 8 (10.1) | 6 (9.2) | 0.97 (0.33-2.88) | 0.953 |

^a^elderly Rivaroxaban group vs. elderly Warfarin group

LVT: left ventricular thrombus

**Table S11:**

clinical covariates included in the multivariate Cox proportional hazard regression model for each outcome

| **clinical covariates included in the multivariate Cox proportional hazard regression model for each outcome** |
| --- |
| **Model 1**  ***Major adverse cardiovascular events:*** LV ejection fraction, NT-proBNP, Diabetes mellitus, Current smoker, Creatinine clearance |
| **Model 2**  ***LV thrombus resolution:*** Types of anticoagulation, LV ejection fraction, white blood cell |
| **Model 3**  ***All-cause mortality:*** LV ejection fraction, NT-proBNP, Types of anticoagulation |
| **Model 4**  ***Systemic embolism:*** Prior SSE, Antiplatelet therapy, Types of anticoagulation |
| **Model 5**  ***Rehospitalization for cardiovascular events:*** Current smoker |
| **Model 6**  ***Bleeding events:*** For this outcome, due to the number of events, so this outcome was finally assessed by a Cox proportional hazard regression model according to the EPV=10 principle. |
| **Noted:** According to clinical weights of covariates and the EPV=10 principle, covariates with P≤0.05 in the univariate models were included in the multivariate Cox proportional hazards regression models to identify the independent effect of anticoagulant types on outcomes. |

**Figure S1:**

Subgroup Analysis of LVT resolution

| **Subgroup Analysis of LVT resolution**  **Elderly LVT group VS. Younger LVT group** |
| --- |
| 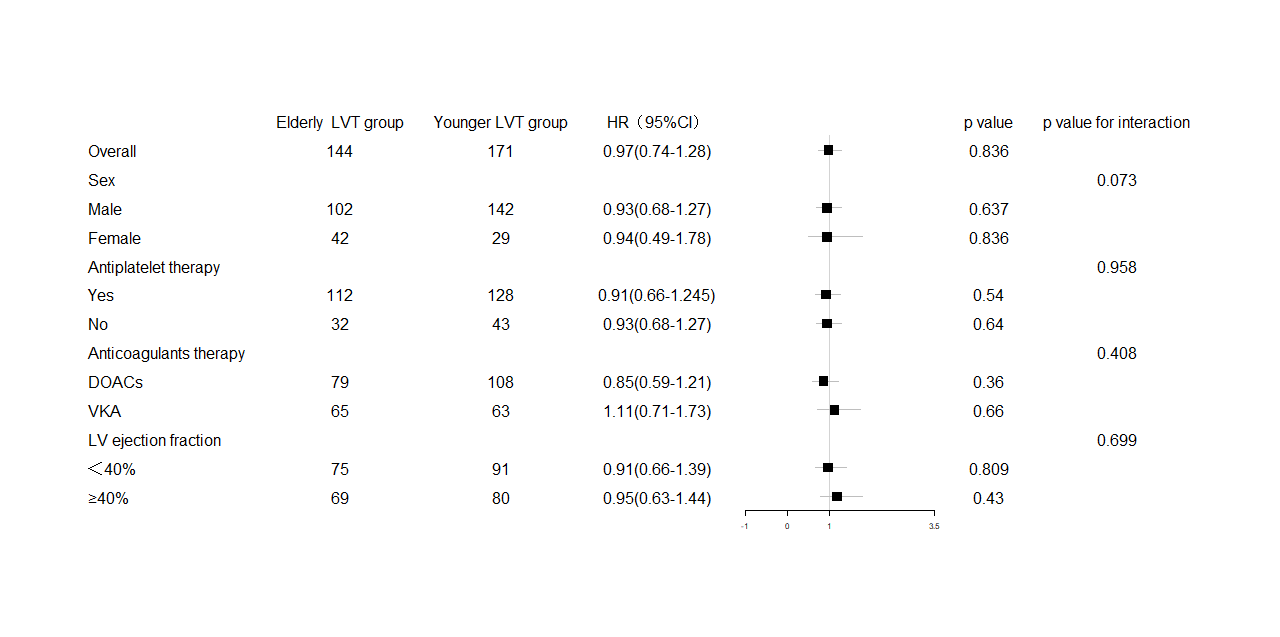 |
| LVT: left ventricular thrombus; LV ejection fraction: left ventricular ejection fraction; DOACs: direct oral anticoagulants; VKA: vitamin K antagonist  Note: P value for Interaction based on the Cox proportional Hazard joint test. |

**Figure S2:**

Subgroup Analysis of Major Adverse Cardiovascular Events

| **Subgroup Analysis of MACE**  **Elderly LVT group VS. Younger LVT group** |
| --- |
| 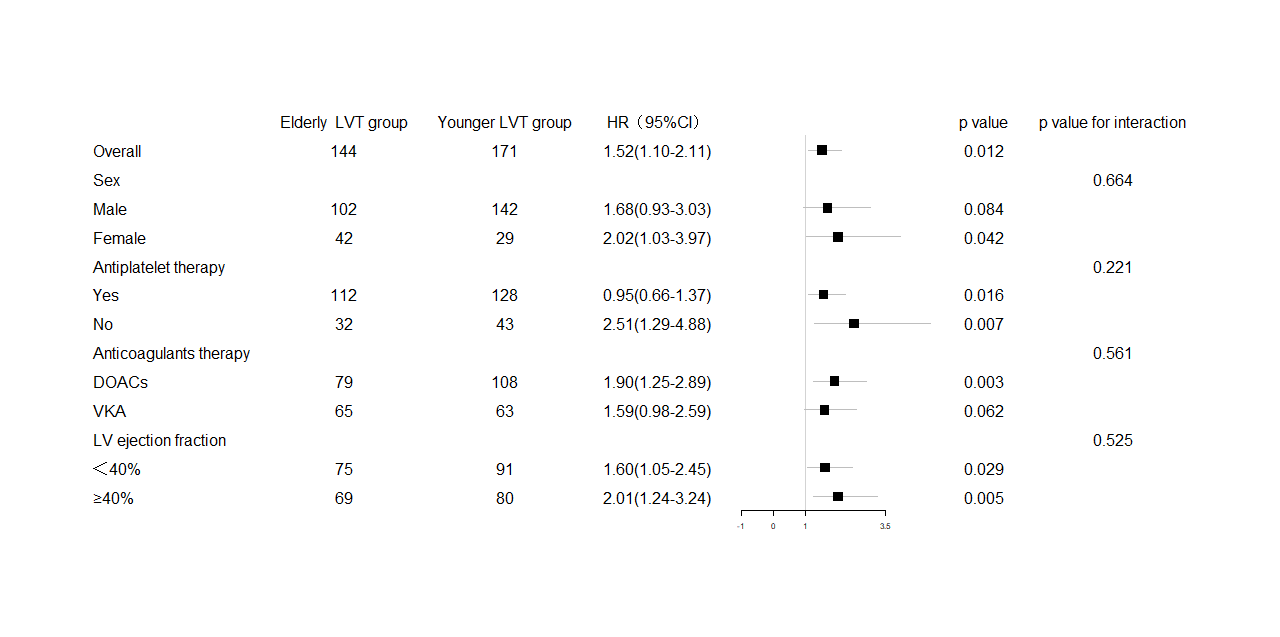 |
| LVT: left ventricular thrombus; LV ejection fraction: left ventricular ejection fraction; DOACs: direct oral anticoagulants; VKA: vitamin K antagonist; MACE: Major adverse cardiovascular event  Note: P value for Interaction based on the Cox proportional Hazard joint test. |

**Figure S3:**

Subgroup Analysis of Rehospitalization for Cardiovascular Events

| **Subgroup Analysis of Rehospitalization for Cardiovascular Events**  **Elderly LVT group VS. Younger LVT group** |
| --- |
| 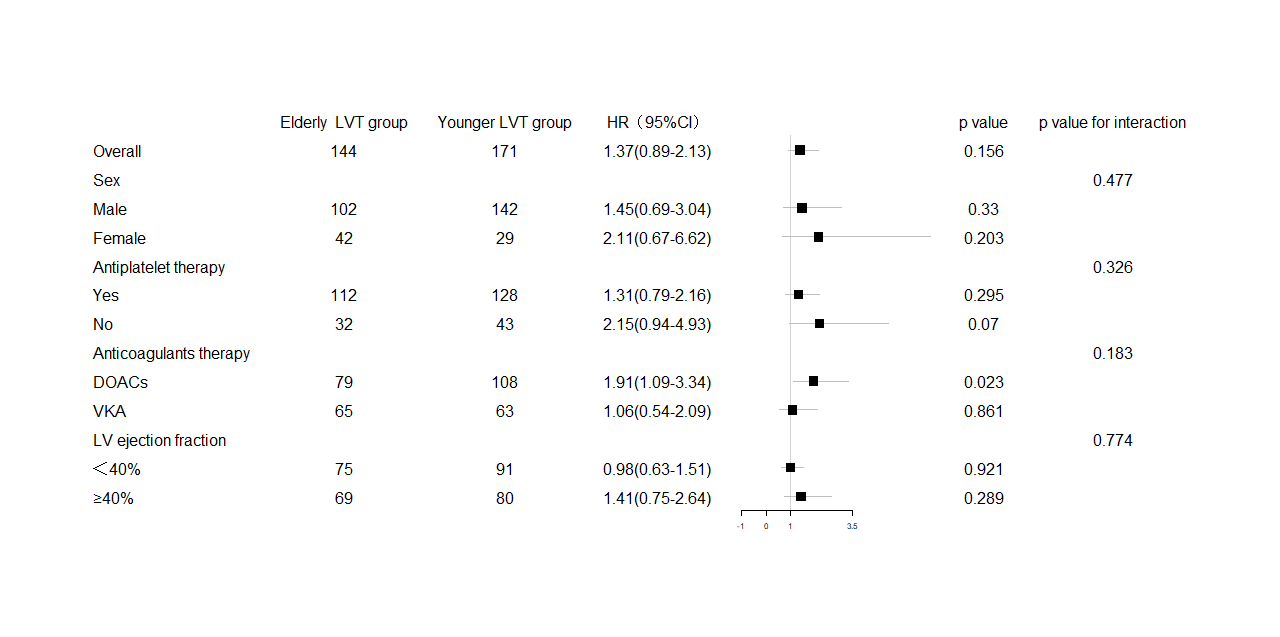 |
| LVT: left ventricular thrombus; LV ejection fraction: left ventricular ejection fraction; DOACs: direct oral anticoagulants; VKA: vitamin K antagonist  Note: P value for Interaction based on the Cox proportional Hazard joint test. |

**Figure S4:**

Subgroup Analysis of Systemic Embolism

| **Subgroup Analysis of Systemic Embolism**  **Elderly LVT group VS. Younger LVT group** |
| --- |
| 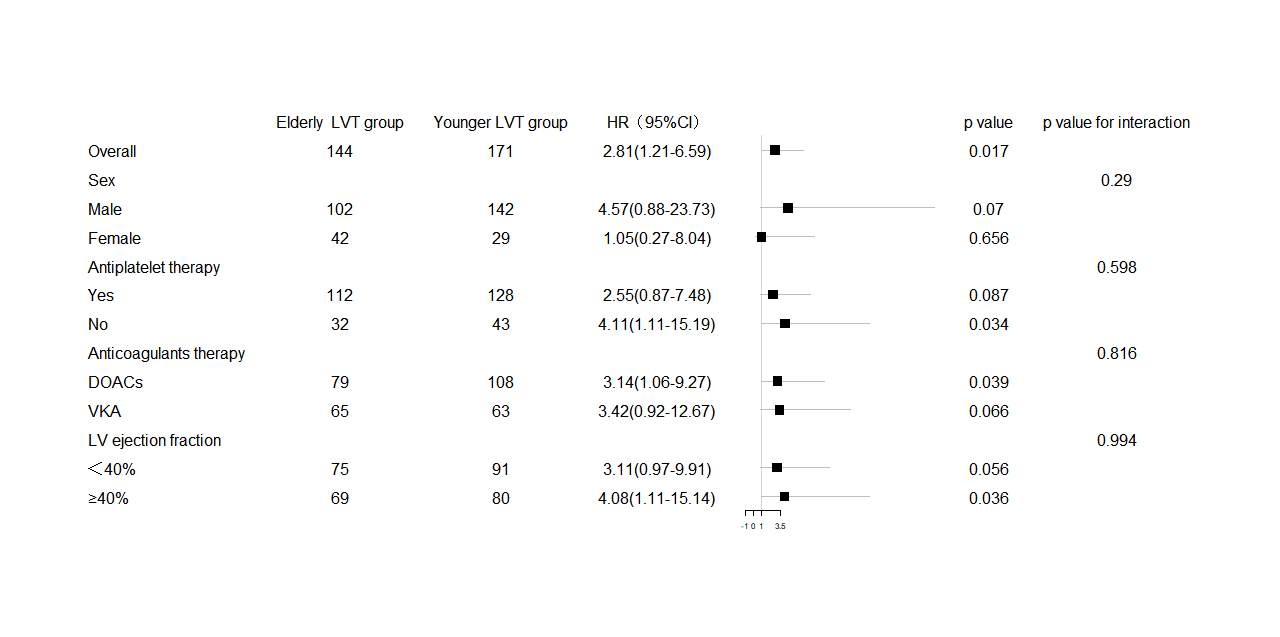 |
| LVT: left ventricular thrombus; LV ejection fraction: left ventricular ejection fraction; DOACs: direct oral anticoagulants; VKA: vitamin K antagonist  Note: P value for Interaction based on the Cox proportional Hazard joint test. |

**Figure S5:**

Subgroup Analysis of All-cause Mortality

| **Subgroup Analysis of all cause of mortality**  **Elderly LVT group VS. Younger LVT group** |
| --- |
| 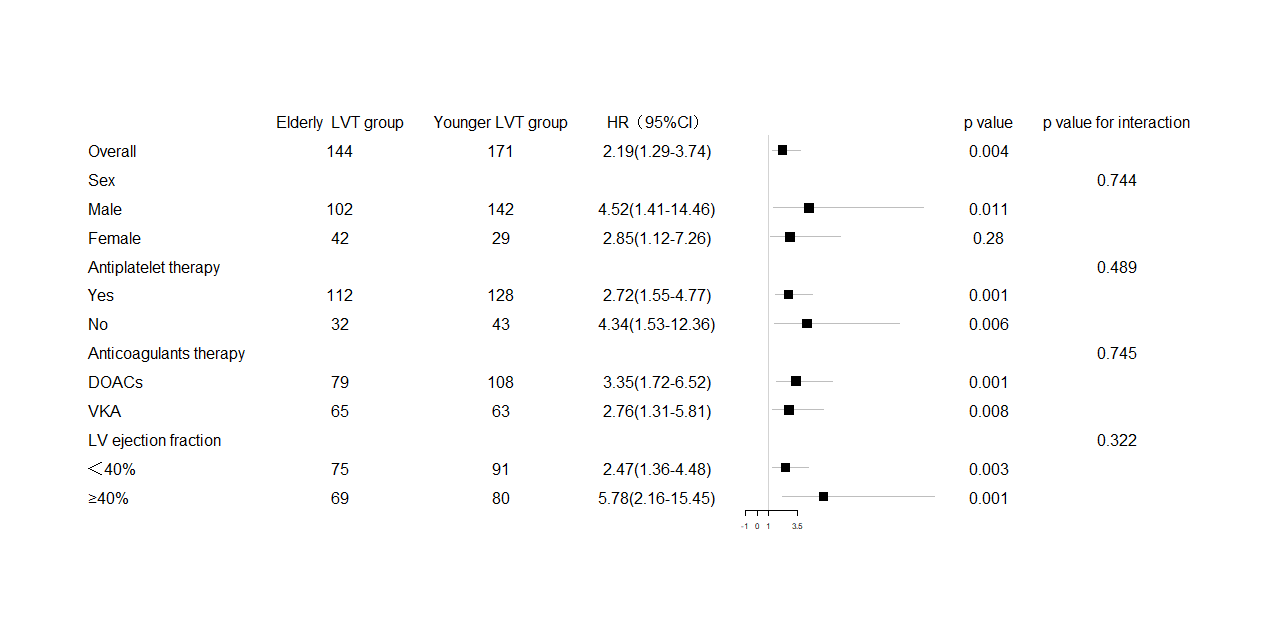 |
| LVT: left ventricular thrombus; LV ejection fraction: left ventricular ejection fraction; DOACs: direct oral anticoagulants; VKA: vitamin K antagonist  Note: P value for Interaction based on the Cox proportional Hazard joint test. |
